# Supplementary material for: Social determinants of COVID-19 mortality at the county level
Source: PLoS One. 2020 Oct 14;15(10):e0240151. doi: 10.1371/journal.pone.0240151 (PMC7556498; doi:10.1371/journal.pone.0240151)
Supplement: S1 File — (DOCX) [file pone.0240151.s001.docx]

PONE-D-20-18322R1
Social determinants of COVID-19 mortality at the county level
PLOS ONE

Dear Dr. Fielding-Miller,

Thank you for submitting your manuscript to PLOS ONE. While you have thoughtfully addressed many of the previous reviewer comments, some concerns remain. Therefore, we feel that your manuscript does not fully meet PLOS ONE’s publication criteria as it currently stands. We invite you to submit a revised version of the manuscript that addresses the points raised during the review process.

One of the more important concerns relates to the modeling approach and that there is a lack of detail regarding how the authors addressed (or did not address) the fact that a quarter of counties had a mortality equal to 0. There are multiple statistical approaches that could be used to address potential modal instability due to a relatively large number of 0s. At the very least, this should be further discussed in the limitations section of the manuscript.

We have addressed this potential shortcoming under Reviewer 1, Comment 2.


**Comments to the Author**


Reviewer #1: This paper evaluates the association of COVID-19 mortality with social determinants at the county level. The authors have taken great efforts to address the comments by the reviewers, and the paper remains important in that it highlights aspects related to the COVID-19 pandemic. A few questions regarding method choices should be addressed as outlined below.

Major comments

1. The authors have taken the reviewer’s suggestion to evaluate rate of mortality per 100,000 residents stratified by census region. I suggest that the findings from this analysis be provided as well.

These findings are presented in lines 154 – 170. We present these findings by region rather than nationally because the national level findings would mask the significant variation by region.

Relatedly, I’m unsure if the information in lines 189-192 means overall or in the regional analysis. This could be a reasonable place to note the overall analysis per 100,000 in an appendix.

We have added language to this section to clarify that we are discussing sub-analyses. The line now reads as follows:

*Across all 9 census regions, there was no association between the percentage of farmworkers and mortality…*

2. The authors should clearly address limitations of their study and what implications the limitations may have. For example, 25% of the counties included in the analysis have 0 deaths. With a highly skewed outcome (as evident by the range versus the IQR and median) it is important to highlight what this may mean in terms of the analysis using spatial autoregressive models. Second, there should be a discussion about the range of the primary variable of focus (percent of farmers) among urban counties, which has an IQR of 0.0 to 0.1. This may be related to the finding of an increase of 2,500 deaths for each 1 percentage point increase in urban counties. It is likely that this may not be a stable model.

We appreciate the reviewer’s thoughtfulness. We agree, these data are highly non-normal and careful attention to model building is necessary. We have added the following language to the text regarding our approach:

*Our spatial autoregressive model used the generalized spatial two-stage least squares estimator, specifically Stata’s spregress command with the “g2sl” option. G2sl is a generalized method of moment (GMM) modeling approach. The GMM makes no assumptions about variable distribution and is an appropriate statistical approach to address data that are highly skewed or have unknown distributions (15).*

3. Figures 4 and 5 may be a bit challenging to interpret without more context. Potentially change the words “percentage” to “a one percentage point change in.”

We have revised the figures and hope they are now more clear.

4. I am a bit confused on the map legends in Figure 3. There are multiple categories that show 0.0-0.0, which likely are extra categories or simple mislabeling/typos. However, I am unsure how the percent of residents in poverty are all in the hundreds or over 1,000.

Thank you, some of these were errors from the software, others were typos. We have revised figure 3 extensively for clarity.

Minor comments

1. There remains two more uses of “more” rather than “percent of” in the abstract and in the last sentence of the introduction. I suggest modifying these for consistency and accuracy

2. Median death count on line 103 does not match what is in table 1. Text median = 2, and table median = 6.

Thank you for catching this oversight. We have corrected the text.

3. The population (thousands) variable is flipped between non-urban and urban counties in table 1.

Thank you! We appreciate the reviewer’s attention to detail. This has been corrected

4. In table 2, please clarify that these outcomes are related to the death counts analysis (rather than per 100,000). Potentially include a footer to explain what b-direct/indirect/total mean for the reader.

We appreciate this suggestion and agree that it will enhance the paper’s clarity.

5. The highest end of the largest level in the legend in Figure 2 (1615.6) does not match the maximum given in the text 350).

We have modified all figures to address the reviewer’s comments.

Reviewer #2: Thank you for taking time to make a powerful analysis even sharper.

One thing:
Something off with line 186: "Even when adjusting for the percentage of non-186 English speaking households, percentage of people living in poverty, percentage of people without insurance, and the percentage of farmworkers in a county appears to be independently associated with a higher number of deaths, and that the relationship varies by region."*.*

We have modified this sentence and believe it now reads more clearly.
